# Incidence and impact of interstitial lung disease and malignancy in patients with polymyositis, dermatomyositis, and clinically amyopathic dermatomyositis: a retrospective cohort study

**DOI:** 10.1186/s40064-015-1013-8

**Published:** 2015-05-28

**Authors:** Satoshi Ikeda, Machiko Arita, Kenta Misaki, Shohei Mishima, Takuya Takaiwa, Akihiro Nishiyama, Akihiro Ito, Kenjiro Furuta, Toshihide Yokoyama, Fumiaki Tokioka, Maki Noyama, Hiroshige Yoshioka, Tadashi Ishida

**Affiliations:** Department of Respiratory Medicine, Kurashiki Central Hospital, Miwa 1-1-1, Kurashiki City, Okayama 710-8602 Japan; Department of Rheumatology, Kurashiki Central Hospital, Miwa 1-1-1, Kurashiki City, Okayama 710-8602 Japan

**Keywords:** Interstitial lung disease, Malignancy, Polymyositis, Dermatomyositis, Clinically amyopathic dermatomyositis

## Abstract

The aims of this study were to retrospectively review Japanese consecutive cases of polymyositis (PM), dermatomyositis (DM), and clinically amyopathic dermatomyositis (CADM), focusing on interstital lung disease (ILD) and malignancy, and to document any differences in the incidence, clinical features, and impact on prognosis among patients with PM, DM, and CADM.

We retrospectively reviewed 62 consecutive patients diagnosed with PM, DM, and CADM according to Bohan and Peter’s criteria (PM/DM) and Sontheimer’s criteria and Gerami’s criteria (CADM), focusing on ILD and malignancy.

ILD occurrence rates were 48 % (11/23) in patients with PM, 46 % (11/24) in DM, and 100 % (15/15) in CADM. Malignancy occurred during diagnosis or the observation period in 14 patients; 86 % were without ILD, and 64 % were DM without ILD. Multivariate logistic regression analysis showed that the risk of newly diagnosed malignancy was significantly lower in patients with ILD [odds ratio, 0.0688; 95 % confidence interval (CI), 0.00127–0.372; *p* = 0.00190] and significantly higher in patients with DM (odds ratio, 5.21; 95 % CI, 1.17–23.1; *p* = 0.0299) than in other patients. Patients with malignancies had shorter survival than those without malignancies; no clinically meaningful difference in survival was observed among the different myositis types and for presence of ILD. In CADM-ILD, 80 % fatal cases died from refractory ILD ≤90 days from the first visit; neither death nor recurrence occurred subsequently.

In conclusion, a positive association between DM and malignancy and a negative association between ILD and malignancy were noted. In the present study, malignancy was a predictor of poor long-term prognosis, but ILD were not. ILD associated with CADM contributed greatly to poor short-term prognosis, but neither death nor recurrence occurred subsequently.

## Introduction

Polymyositis (PM) and dermatomyositis (DM) are classified as idiopathic inflammatory myopathies characterized by proximal skeletal muscle weakness and muscle inflammation. Bohan and Peter’s criteria, which were published in 1975, are often used for diagnosis (Bohan and Peter 1975). Unlike PM, DM is associated with a variety of characteristic rashes, such as the heliotrope rash and Gottron’s papules. Moreover, in 2002, Sontheimer proposed the concept of clinically amyopathic dermatomyositis (CADM) which has the typical rash of DM but little or no evidence of myositis (Sontheimer 2002).

Interstitial lung disease (ILD) is an important complication in patients with PM, DM, and CADM. The occurrence of ILD has varied widely from 20 to 80 % among case series of patients with PM and DM (Marie et al. 2002; Love et al. 1991; Fathi et al. 2008; Connors et al. 2010). The clinicopathological features and prognosis of ILD vary depending on the type of myositis and myositis-specific antibodies (Fujisawa et al. 2005). ILD associated with CADM is often refractory and rapidly progressive (Douglas et al. 2001). The ratio of rapidly progressive ILD to the total population of CADM-ILD has been reported to be higher in eastern Asia than in Europe and the US. Although the presence of anti-aminoacyl tRNA synthetase (anti-ARS) antibodies, such as anti Jo-1 antibody, is associated with chronic ILD, anti-MDA5 antibodies are associated with acute/subacute ILD (Sato et al. 2012, 2013).

Moreover, malignancies are also associated with inflammatory myopathies. Several population-based cohort studies have confirmed the increased risk of malignancy among patients with PM and DM (Sigurgeirsson et al. 1992; Buchbinder et al. 2001; Stockton et al. 2001). The close relationship between malignancy and inflammatory myopathy may be because of the paraneoplastic processes caused by the expression of common autoantigens in cancer tissue and muscle tissue in some patients with PM and DM (Albert and Darnell 2004; Casciola-Rosen et al. 2005).

Among patients with PM, DM, and CADM, ILD and malignancies have been considered to be a major cause of morbidity and mortality. However, there have been only a few studies on the incidence, clinical characteristics, and clinical course of ILD and malignancy for the different types of myositis. In addition, the effects of ILD and malignancy as complications on the outcomes in patients with PM, DM, and CADM are unclear.

The aims of this study were to retrospectively review consecutive Japanese cases of PM, DM, and CADM, focusing on ILD and malignancy, and to document any differences in the incidence, clinical features, and impact on prognosis among PM, DM, and CADM.

## Patients and methods

### Patients and setting

This retrospective study was conducted at Kurashiki central hospital in Kurashiki City, Okayama, Japan. The diagnoses of PM and DM were based on Bohan and Peter’s criteria. The diagnosis of CADM was based on the criteria of Sontheimer (2002). In addition, patients who exhibited cutaneous manifestations of DM without muscle weakness for <6 months and experienced fatal complications, such as acute or subacute ILD, were also diagnosed with CADM according to Gerami’s criteria (Gerami et al. 2006). From January 2005 to December 2012, 62 cases newly diagnosed with PM, DM, and CADM by two or more physicians (including a rheumatologist) were included in the study. There were no exclusion criteria. The patients were divided into five groups depending on the type of myositis and presence of ILD: PM with ILD (PM-ILD; 11 cases), PM without ILD (PM-no ILD; 12 cases), DM with ILD (DM-ILD; 11 cases), DM without ILD (DM-no ILD; 13 cases), and CADM with ILD (CADM-ILD; 15 cases). No cases of CADM without ILD were present. The Ethics Committee of Kurashiki Central Hospital approved this study protocol.

### Clinical and laboratory findings

The clinical and laboratory data used in the study were based on the patients’ medical records. The factors examined included gender, age, smoking history, time from onset to first visit or treatment start, department for the first visit, symptoms and physical examination, laboratory data, and pulmonary function test results.

### Radiological findings

All of the patients with ILD underwent high-resolution computed tomography (HRCT) at the time of diagnosis. HRCT findings were reviewed and interpreted by two pulmonologists and one radiologist. Images were assessed for the predominant lung zone (upper or lower lobe), predominant distribution (peribronchovascular or subpleural), presence of consolidation, ground-glass opacity, reticular shadow, irregular linear opacity, traction bronchiectasis, cyst, subpleural curve linear shadow, thickening of the interlobular septa, emphysema, and volume loss.

### Statistical analysis

Categorical data are presented as numbers (percentages). Continuous data are presented as the median (interquartile range). Fisher’s exact test was used to compare categorical data. The Mann–Whitney *U*-test was used to compare continuous data. Univariate and multivariate logistic regression analysis was performed to verify the risk of newly diagnosed malignancy. Cumulative survival probabilities were estimated using the Kaplan–Meier method. A *p* value of <0.05 was considered statistically significant.

### Ethics approval

Ethics approval was provided by the Ethics Committee of Kurashiki Central Hospital.

## Results

### Characteristics

ILD was found in 37 cases (60 %). The ILD occurrence rates were 48 % (11/23) in patients with PM, 46 % (11/24) in patients with DM, and 100 % (15/15) in patients with CADM. No significant differences were observed in gender, age, and smoking history among the five groups (Table 1). The median time from the onset of symptoms to first visit or therapy initiation tended to be short in the patients with ILD, especially those in the CADM-ILD group. The Department of Respiratory Medicine was usually the first department in which the diagnosis of ILD was made in the PM-ILD and CADM-ILD groups. The Department of Rheumatology was most frequently visited in the DM group regardless of ILD diagnosis.

**Table 1:** Summary of the clinical characteristics and laboratory data

	PM		DM		CADM
	ILD (*N* = 11)	No ILD (*N* = 12)	ILD (*N* = 11)	No ILD (*N* = 13)	ILD (*N* = 15)
Sex (male/female)	2/9	5/7	2/9	4/9	5/10
Age (%)	66.0 (58.0–72.5)	68.5 (62.3–71.3)	65.0 (53.5–68.0)	68.0 (51.0–78.0)	63.0 (60.5–69.0)
Smoking history (%)	1 (9 %)	6 (50 %)	3 (27 %)	2 (15 %)	6 (40 %)
Days from onset					
To 1st visit	44.0 (14.0–109)	184 (59.0–761)	68.0 (29.5–96.5)	97.0 (63.0–238)	17.0 (9.50–38.5)
To treatment	62.0 (24.0–151)	235 (133–902)	80.0 (50.3–175)	127 (108–282)	31.0 (15.5–100)
Department for the first visit (%)					
Respiratory medicine	8 (73 %)	0	4 (36 %)	0	9 (60 %)
Rheumatology	3 (27 %)	4 (33 %)	6 (55 %)	8 (62 %)	4 (27 %)
Neurology	0	8 (67 %)	1 (9 %)	3 (23 %)	0
Dermatology	0	0	0	2 (15 %)	2 (13 %)
Symptom and signs (%)					
Skin					
Gottron’s sign / papule	0	0	6 (55 %)	10 (77 %)	9 (60 %)
Heliotrope eruption	0	0	3 (27 %)	10 (77 %)	8 (53 %)
V / Shawl neck sign	0	1 (8 %)	4 (36 %)	6 (46 %)	4 (27 %)
Mechanic’s hands	0	0	2 (18 %)	1 (8 %)	2 (13 %)
Muscle					
Myalgia	3 (27 %)	5 (42 %)	8 (73 %)	2 (15 %)	4 (27 %)
Muscle weakness	10 (91 %)	12 (100 %)	6 (55 %)	13 (100 %)	0
Lung					
Dry cough	5 (45 %)	0	3 (27 %)	0	9 (60 %)
Dyspnea on exertion	2 (18 %)	1 (8 %)	2 (18 %)	0	9 (60 %)
Others					
Fever	8 (73 %)	1 (8 %)	7 (64 %)	1 (8 %)	12 (80 %)
Laboratory data					
C-reactive protein (mg/dl)	1.06 (0.535–3.28)	0.125 (0.0800–0.320)	0.730 (0.330–1.96)	0.320 (0.110–0.610)	1.27 (0.495–2.92)
Krebs von den Lungen-6 (U/ml)	870 (568–1340)	273 (127–288)	648 (297–1020)	241 (234–289)	905 (596–1197)
Surfactant protein D (ng/dl)	119 (68.8–180)	52.3 (45.2–62.0)	70.1 (45.5–182)	23.1 (17.2–73.3)	64.7 (29.8–117)
Aspartate aminotransferase (IU/l)	85.0 (62.0–150)	51.5 (35.5–100)	66.0 (48.0–157)	68.0 (54.0–158)	40.0 (28.5–56.5)
Alanine aminotransferase (IU/l)	50.0 (31.5–111)	27.0 (16.5–75.0)	50.0 (29.0–79.5)	43.0 (28.0–101)	20.0 (17.0–32.5)
Lactate dehydrogenase (IU/l)	571 (429–772)	317 (263–670)	496 (357–645)	513 (441–759)	342 (283–381
Creatine phosphokinase (IU/l)	1547 (599–2657)	1212 (402–3347)	874 (270–3000)	965 (617–4872)	232 (108–316)
Aldolase (U/l)	28.5 (11.1–45.2)	18.1 (5.15–22.8)	15.5 (7.75–31.9)	11.4 (8.40–30.9)	7.40 (3.45–8.95)
Antinuclear antibody (%)	8 (73 %)	7 (58 %)	7 (64 %)	9 (69 %)	8 (53 %)
Anti-Jo-1 antibody (%)	1 (9 %)	0	1 (9 %)	1 (8 %)	0
Anti-MDA5 antibody	NA	NA	NA	NA	8/13
Lung function test					
%Forced vital capacity	76.5 (59.8–93.8)	NA	76.5 (53.0–81.7)	NA	83.1 (70.4–91.3)
%Diffusing capacity for carbon monoxide	65.7 (56.0–73.6)	NA	61.3 (47.1–64.0)	NA	58.2 (54.6–62.4)

Categorical data are presented as numbers (percentages). Continuous data are presented as the median (interquartile range). *Abbreviations*: NA, not applicable

Regarding skin manifestations, Gottron’s sign was the most common in the patients with DM (with or without ILD) and CADM, and no statistically significant differences were observed among the three groups regarding exhibition of the typical rash of DM. Muscle weakness was not observed in the CADM-ILD group, whereas myalgia was observed in four of 15 cases (27 %) with CADM-ILD. Respiratory symptoms (dry cough and dyspnea on exertion) and fever were more frequently observed in the CADM-ILD group than in the other two groups with ILD, but the differences were not statistically significant.

In the CADM-ILD group, no increase in myogenic enzymes, such as CPK and aldolase, was observed relative to those in the other four groups. No differences were observed in the C-reactive protein, ILD biomarkers, and pulmonary function test results among the three groups with ILD. Although all patients were examined, anti-Jo-1 antibody was detected in only three patients. Anti-MDA5 antibody was detected in eight of 13 patients examined in the CADM-ILD group.

### HRCT findings of ILD

In patients with ILD in all groups, shadows were predominantly observed in the lower lobe (Table 2). In the PM-ILD group, peri-bronchovascular consolidation, suggestive of cellular nonspecific interstitial pneumonia (NSIP), was most frequently observed. On the other hand, the imaging findings of the DM-ILD group were relatively diverse. The NSIP pattern and organizing pneumonia patterns were most frequently suspected. In the CADM-ILD group, subpleural consolidation or irregular linear opacity was typically observed. Although HRCT findings of CADM-ILD were often seemingly mild, many of them were associated with significant volume loss in the lower lobes or with traction bronchiectasis. Subpleural curve linear shadow suggestive of collagen vascular disease was found in 36 %, 64 %, and 47 % of the patients in the PM-ILD, DM-ILD, and CADM-ILD groups, respectively.

**Table 2:** Comparison of high-resolution computed tomography findings among the patients with polymyositis, dermatomyositis, and clinically amyopathic dermatomyositis

	PM-ILD (*N* = 11)	DM-ILD (*N* = 11)	CADM-ILD (*N* = 15)	*p*-value (adjusted by Holm method)		
				PM vs DM	PM vs CADM	DM vs CADM
Distribution						
Upper lobe dominant	0	1 (9 %)	0	1.00	1.00	1.00
Lower lobe dominant	11 (100 %)	11 (100 %)	15 (100 %)	-	-	-
Peri-bronchovascular	9 (82 %)	5 (45 %)	6 (40 %)	0.370	0.150	1.00
Subpleural	2 (18 %)	7 (64 %)	11 (73 %)	0.161	0.0460	0.683
Shadow						
Consolidatiuon	7 (64 %)	4 (36 %)	7 (47 %)	1.00	1.00	1.00
Ground glass opacity	1 (9 %)	3 (27 %)	3 (20 %)	1.00	1.00	1.00
Reticular shadow	3 (27 %)	5 (45 %)	2 (13 %)	1.00	1.00	0.280
Irregular linear opacity	0	0	4 (27 %)	1.00	0.340	0.340
Tractionbronchiectasis	9 (82 %)	7 (64 %)	10 (67 %)	1.00	1.00	1.00
Cyst	0	1 (9 %)	1 (7 %)	1.00	1.00	1.00
Subpleural curve linear shadow	4 (36 %)	7 (64 %)	7 (47 %)	1.00	1.00	1.00
Interlobular septa thickening	1 (9 %)	2 (18 %)	4 (27 %)	1.00	1.00	1.00
Others						
Emphysema	2 (18 %)	1 (9 %)	2 (13 %)	1.00	1.00	1.00
Volume loss	9 (82 %)	6 (55 %)	13 (87 %)	0.720	1.00	0.280

Categorical data are presented as numbers (percentages) and were analyzed by Fisher’s exact test, adjusted by the Holm method

### Malignancy as a complication

Malignancy as a complication was observed in 17 patients (Table 3). Three of these patients had past histories of surgical resection for malignancy, and no recurrences were subsequently observed. Fourteen patients were newly detected during the diagnosis of PM, DM, and CADM or during the observation period, 86 % of whom were patients without ILD, and 64 % were in the DM-no ILD group. Among the 14 patients with newly diagnosed malignancy, nine had metastatic and five had localized malignancies. The most common site of origin was gastric cancer (three patients), and the most common type of cancer was adenocarcinoma (five patients).

**Table 3:** Complication of malignancy

(A)	PM		DM		CADM
	ILD (*N* = 11)	No ILD (*N* = 12)	ILD (*N* = 11)	No ILD (*N* = 13)	ILD (*N* = 15)
Past history					
Number of patients	1	1	0	0	1
Primary site	gastric (ad)	lung (ad)			prostate (ad)
Newly diagnosed					
Number of patients	0	3	1	9	1
Primary site (histology)		CML	breast (meta)	nasopharyngeal (sq)	ovarian (clear)
		ML (MALT)		lung (sq;1, ad;1)	
		colorectal (ad)		gastric (ad;3)	
				oropharyngeal (sq)	
				ML (DLBCL)	
				Thymoma (typeB3)	
(B)	Odds ratio (95 % CI)		*P* value		
Univariate analysis					
PM	0.387 (0.0615–1.73)		0.218		
DM	5.87 (1.41–30.2)		0.0106		
CADM	0.191 (0.00411–1.51)		0.155		
ILD	0.0652 (0.00628–0.350)		0.000126		
Multivariate analysis					
DM	5.21 (1.17–23.1)		0.0299		
ILD	0.0688 (0.0127–0.372)		0.00190		

Details of complicated malignancies (A); univariate and multivariate logistic regression analysis verified the risk of newly diagnosed malignancy (B)

*ad* adenocarcinoma, *sq* squamous cell carcinoma, *meta* metaplastic carcinoma, *clear* clear cell, *CML* chronic myelogenous leukemia, *ML* malignant lymphoma, *MALT* mucosa-associated lymphoid tissue, *DLBCL* diffuse large B-cell lymphoma

Multivariate logistic regression analysis showed that the risk of newly diagnosed malignancy was significantly lower in the patients with ILD (odds ratio, 0.0688; 95 % confidence interval (CI), 0.00127–0.372; *p* = 0.00190) and significantly higher in the patients with DM (odds ratio, 5.21; 95 % CI, 1.17–23.1; *p* = 0.0299).

### Treatment

In the patients with PM (with or without ILD) and DM-no ILD, monotherapy with corticosteroids was the most common initial treatment (Table 4), whereas six of the 11 patients (55 %) with DM-ILD received combination therapy with corticosteroids and some kind of immunosuppressant. In the CADM-ILD group, initial treatment consisted of a three-drug combination therapy with corticosteroids, cyclosporine and cyclophosphamide in six patients, and combination therapy with corticosteroids and cyclosporine in six cases. In addition, the initial treatment consisted of combination therapy with corticosteroid and cyclophosphamide, monotherapy with corticosteroid, and no treatment in one patient each.

**Table 4:** Treatment and outcomes

	PM		DM		CADM
	ILD (*N* = 11)	No ILD (*N* = 12)	ILD (*N* = 11)	No ILD (*N* = 13)	ILD (*N* = 15)
Initial immunosuppressive therapy					
Prednisone + Cyclosporine + Cyclophosphamide	1	0	1	0	6
Prednisone + Cyclosporine	1	0	2	0	6
Prednisone + Cyclophosphamide	0	0	1	0	1
Prednisone + Azathioprine	1	3	1	2	0
Prednisone + Tacrolimus	0	0	1	0	0
Prednisone + Methotrexate	0	2	0	1	0
Prednisone	8	5	4	9	1
No therapy	0	2	1	1	1
Treatment for maligancy					
Surgical resection	0	1	1	4	0
Chemotherapy	0	2	1	4	0
Outcome					
Death (number of patients)	1	3	4	5	5
Days from first visit to death	267	1520 (1179–2125)	376 (232–738)	122 (32–1071)	33 (27–41)
Cause of death	Renal failure	Aspiration pneumonia	ILD (2)	Aspiration pneumonia (3)	ILD (4)
		Hypoglycemia	Breast cancer	Malignant lymphoma	Extradural hematoma
		Sepsis	Brainstem infarction	lung cancer	

Initial immunosuppressive treatment and treatment for malignancies diagnosed simultaneously with the diagnoses of polymyositis, dermatomyositis, and clinically amyopathic dermatomyositis or during the observation period and outcomes

Treatment for the 14 patients with newly diagnosed malignancies consisted of surgical resection in six patients and chemotherapy in seven. Two of the patients with ILD had malignancies; one patient with DM-ILD had metastatic breast cancer, and although she underwent surgery and received chemotherapy (epirubicin, cyclophosphamide, and docetaxel as an adjuvant chemotherapy; S-1 and letrozole for the recurrent cancer), she died from progression of breast cancer. Another patient with CADM-ILD had metastatic ovarian cancer, but she died from an extradural hematoma caused by head banging before treatment initiation.

### Outcomes

The median follow-up period for all patients was 48.2 months (the cut-off date for data collection was September 18, 2014). In this study, 18 fatal cases were observed during the follow-up period (one patient in the PM-ILD, three patients in the PM-no ILD, four patients in the DM-no ILD group, five patients in the DM-ILD group, and five patients in the CADM-ILD group) (Table 4). For the whole population, the most common cause of death was ILD (33 %; two patients in the DM-ILD group and four patients in the CADM-ILD group), followed by aspiration pneumonia (22 %) and malignancy (17 %). Among the patients with ILD, ILD was the most common cause of death in the patients with DM and CADM (50 % and 80 %, respectively), whereas no patient with PM died from ILD. In the patients without ILD, aspiration pneumonia caused by respiratory muscle weakness was the most common cause of death. All three patients who died from malignancy had DM.

In the CADM-ILD group, 80 % (four patients) of the patients who were fatal cases died from refractory ILD ≤90 days from their first visit and were positive for anti-MDA5 antibody. In the four patients who died from ILD, the initial treatments were combination therapy with three drugs (corticosteroids, cyclosporine, and cyclophosphamide) for one patient, combination therapy with corticosteroids and cyclosporine for two patients, and corticosteroids for one patient.

**Fig. 1:**
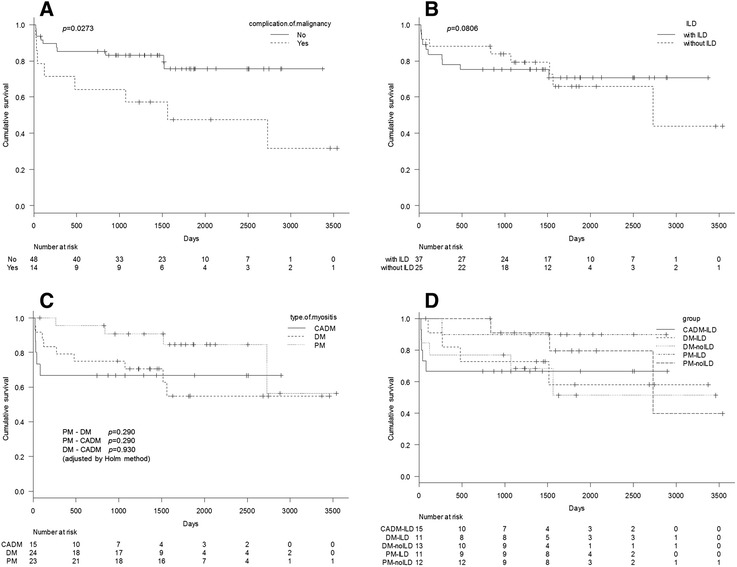
Survival curves for the whole population. Comparison of the survival curves with and without malignancy (**a**), with and without interstitial lung disease (*ILD*) (**b**), by the types of myositis (**c**), by the types of myositis, and by presence or absence of ILD (**d**)

**Fig. 2:**
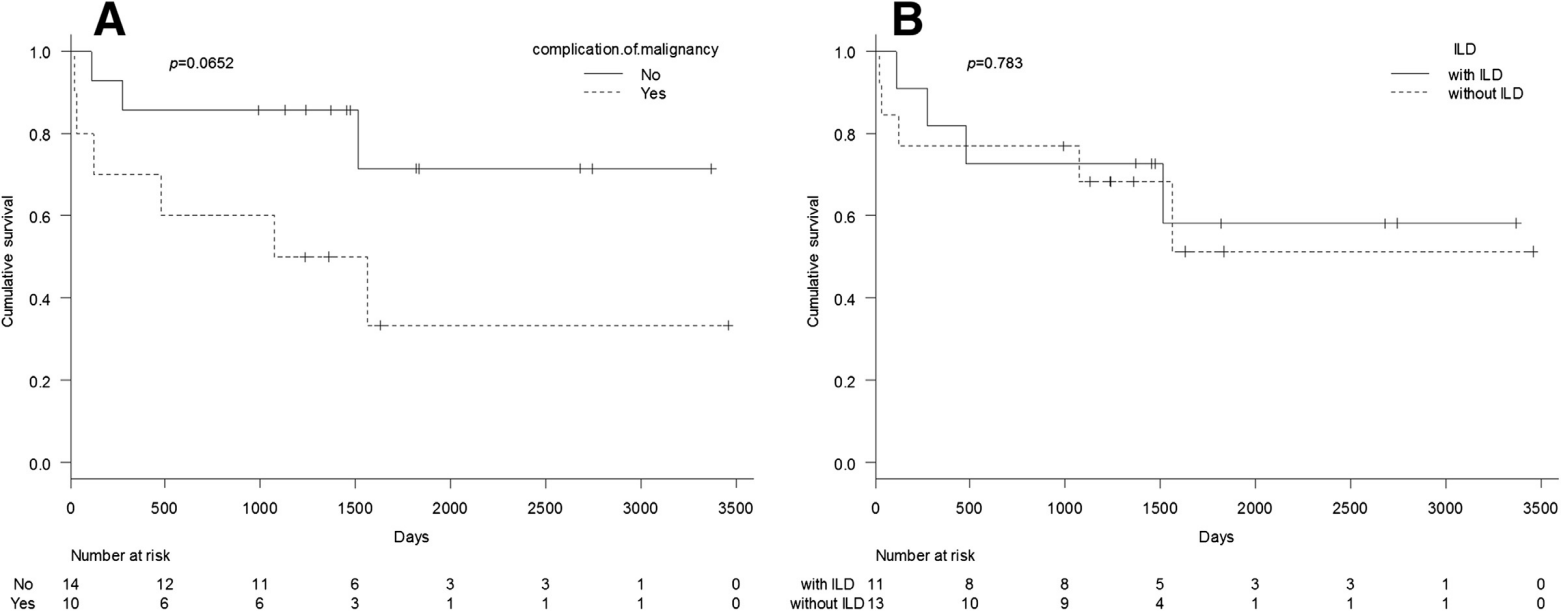
Survival curves for the patients with dermatomyositis. Comparative investigation of the survival curves with and without malignancy (**a**) and with and without interstitial lung disease (**b**)

A comparison of the survival curves is shown in Fig. 1. The patients with malignancies had a shorter duration of survival than did those without malignancies. The median overall survival was 1924 days (95 % CI, 41–not applicable) in the patients with malignancies, whereas the median overall survival in the patients without malignancies was immature at the cut-off date. On the other hand, no clinically meaningful differences were observed in survival among patients with the different types of myositis and for presence or absence of ILD. In a comparison of the five groups, the survival curve showed an initial rapid drop in the CADM-ILD group. However, neither death nor recurrence was observed subsequently in the CADM-ILD group. For the patients with DM, those with malignancies tended to have shorter duration of survival than those without malignancies, but the difference was not statistically significant (Fig. 2).

## Discussion

In this study, we retrospectively reviewed the medical records of consecutive patients with PM, DM, and CADM, focusing on ILD and malignancy, to determine their differences in occurrence, clinical features, and impact on prognosis.

No clinically meaningful difference in overall survival was observed between presence or absence of ILD in this study. However, ILD was the most prevalent cause of death in both the DM-ILD and CADM-ILD groups. In the DM-ILD group, 50 % of the patients in the fatal cases died from ILD. One patient received three-drug combination therapy; however, the patient was refractory and died 111 days after the first visit. In another patient, ILD initially improved following combination therapy with corticosteroids and cyclophosphamide, but relapsed and rapidly progressed during gradual decrease of corticosteroids, which led to death on the 1514th day after the first visit. In the CADM-ILD group, 80 % of the patients in the fatal cases died from refractory ILD ≤90 days from the first visit. On the other hand, in the PM-ILD group, although 73 % of the patients were treated with corticosteroid monotherapy as an initial treatment, no death from ILD occurred. No obvious differences in the laboratory data or lung function test results were observed among the PM-ILD, DM-ILD, and CADM-ILD groups at the first visit. However, patients with ILD complicated with PM, most of which exhibited the radiological cellular NSIP pattern, may have a favorable treatment response. According to past studies, PM-ILD is more responsive to corticosteroid therapy, which results in a favorable prognosis compared with DM-ILD and CADM-ILD (Fujisawa et al. 2005, 2014).

With regards to CADM, the poor short-term prognosis in the CADM-ILD group observed in this study was consistent with previous reports (Mukae et al. 2009; Ye et al. 2007; Schnabel et al. 2003). However, no clinically meaningful differences in overall survival were observed among the CADM-ILD group and the other four groups. One reason may be that neither death nor recurrence occurred in the patients with CADM-ILD who survived the acute phase of ILD. To put it another way, the prognosis of CADM-ILD may depend on whether prompt and intensive treatment can be started. In this study, although 83 % of the CADM-ILD patients treated with three-drug combination therapy survived, one patient with corticosteroid monotherapy died from ILD. In addition, in the patients initially treated with corticosteroids and cyclosporine, 33 % died from ILD and 17 % needed to add cyclophosphamide later. An effective treatment regimen has not yet been established for this condition, but the results of the present study showed that intense treatment with three-drug combination therapy in the early phase, a treatment that was consistent with previous recommendations (Nawata et al. 1999; Yamasaki et al. 2007), may lead to favorable prognosis. In addition, HRCT findings of CADM were often seemingly mild and hard to detect, so it is important to suspect and detect CADM-ILD in conjunction with the typical rash, rapidly progressive respiratory symptoms, and fibrotic findings such as volume loss or traction bronchiectasis in HRCT.

Malignancy as a complication was observed in 14 cases (23 %) during the diagnoses of PM, DM, and CADM or during the observation period in this study. The incidence of malignancy in inflammatory myopathies has been reported to range from 3 to 40 % (Zampieri et al. 2010; Buchbinder and Hill 2002), and adenocarcinoma was the most common histological type of the malignancies associated with inflammatory myopathies (Sato et al. 2013; Buchbinder et al. 2001; Barnes and Mawr 1976; Hill et al. 2001; Bivalacqua et al. 2007; Whitmore et al. 1994), a finding that is consistent with the results of the present study.

In this study, although only three patients died directly from malignancy, the patients with malignancies had a shorter duration of survival than those without malignancy. Previous studies have also suggested that malignancy was a predictive factor of PM/DM deterioration (Callen 2000; Marie et al. 2001). A greater number of deaths caused by malignancy, even in the present study, would probably be observed if a longer follow-up period was used. Although adequate therapy of localized cancer may lead to favorable outcomes for myopathies, 64 % of the malignancies in this study were already metastatic at diagnosis. Thus, prompt investigation and repeated screening for underlying malignancy is very important.

In the present study, multivariate logistic regression analysis showed that the risk of malignancy was significantly higher in the patients with DM than in the other patients. Some population-based studies have also indicated that the risk of cancer was significantly higher in patients with DM than in patients with PM (Antiochos et al. 2009; Airio et al. 1995). Moreover, the risk of malignancy was significantly lower in the patients with ILD. Azuma et al. reported that Japanese patients with PM/DM/CADM who developed malignancies were less likely to have the complication of interstitial lung disease (Azuma et al. 2011). According to a systematic review and meta-analysis, the risk of malignancy was significantly reduced in PM and DM patients who had ILD (risk ratio 0.41; 95 % CI, 0.19–0.87) (Lu et al. 2014). Further studies are needed to elucidate the cause of the negative association between ILD and malignancy in patients with PM/DM, but investigation of specific autoantibodies may contribute to elucidating this mechanism. Although anti-MDA5 antibodies have been strongly associated with rapidly progressive ILD, anti-p155/140 antibodies have been strongly associated with cancer-associated (juvenile) DM (Kaji et al. 2007; Chinoy et al. 2007).

A limitation of the present study was that the number of patients investigated was small, and the distribution of patients may have been skewed. The occurrence rates of ILD in PM (48 %) and DM (46 %) were consistent with those of previous reports (Marie et al. 2002; Love et al. 1991; Fathi et al. 2008; Connors et al. 2010). On the other hand, ILD was observed in all cases of CADM in this study. Most of the CADM patients in this study visited the Department of Respiratory Medicine because of rapidly progressive respiratory symptoms as a chief complaint, whereas many of the patients with only rash may have had very limited contact with a hospital. Some patients may not have fulfilled the Sontheimer’s criteria because of the short duration of the typical rash.

Among the patients with CADM, not only the prevalence of ILD but also the ratio of patients with rapidly progressive ILD to the total population of CADM-ILD has been reported to be higher in eastern Asia than in Europe and the US (Kang et al. 2005; Yamanishi et al. 1999; Sun et al. 2013; Morganroth et al. 2010). Thus, the results of this study apply to CADM patients in eastern Asia but might not apply to CADM patients in Europe and the US.

In conclusion, a positive association between DM and malignancy and a negative association between ILD and malignancy were noted. In the present study, malignancy as a complication was a predictor of poor long-term prognosis, but ILD were not. ILD associated with CADM contributed greatly to poor short-term prognosis, but neither death nor recurrence occurred subsequently.

## Abbreviations

ILD: Interstital lung disease
PM: Polymyositis
DM: Dermatomyositis
CADM: Clinically amyopathic dermatomyositis
HRCT: High resolution computed tomography
NSIP: Nonspecific interstitial pneumonia
